# Positionspapier zur Prähabilitation in Deutschland

**DOI:** 10.1007/s00104-026-02470-9

**Published:** 2026-03-03

**Authors:** Maria Wobith, Eva-Maria Jacob, Johannes Fleckenstein, Florian Herrle, Christoph Reißfelder, Andreas Schnitzbauer

**Affiliations:** 1 Department of Surgery, National University Hospital Singapore, 5 Lower Kent Ridge Road, 119074 Singapur, Singapur; 2 Stabsstelle Ernährungsmedizin, München Klinik Neuperlach, München, Deutschland; 3 Institut für Sportwissenschaften, Goethe-Universität Frankfurt am Main, Frankfurt am Main, Deutschland; 4 Schmerzzentrum, Klinikum Landsberg am Lech, Landsberg am Lech, Deutschland; 5 Abteilung für Allgemein‑/Viszeralchirurgie, Romed Klinik Prien am Chiemsee, Prien am Chiemsee, Deutschland; 6 Chirurgische Klinik, Universitätsmedizin Mannheim, Medizinische Fakultät Mannheim, Universität Heidelberg, Heidelberg, Deutschland; 7 Klinik für Chirurgie, Knappschaft Kliniken Universitätsklinikum Bochum, Ruhr Universität Bochum, Bochum, Deutschland

**Keywords:** Prähabilitation, Perioperatives Management, Multimodale Intervention, ERAS, Implentierung, Prehabilitation, Perioperative management, Multimodal intervention, ERAS, Implementation

## Abstract

Die Prähabilitation gewinnt als Bestandteil einer modernen, patientenzentrierten perioperativen Versorgung zunehmend an Bedeutung. Ziel dieses Positionspapiers ist es, den aktuellen Stand zur Prähabilitation national und international zu umreißen, bestehende Barrieren zur Implementierung zu beleuchten und praxisnahe Empfehlungen zur Umsetzung zu geben. Insbesondere bei älteren, multimorbiden und onkologischen Patienten können gezielte präoperative Interventionen wie Bewegungstherapie, Ernährungstherapie, psychologische Unterstützung und weitere Maßnahmen dazu beitragen, postoperative Komplikationen zu reduzieren, die Genesung zu beschleunigen und die Lebensqualität zu verbessern. Trotz wachsender internationaler Evidenz ist die Implementierung in Deutschland bislang schleppend und häufig durch fehlende Vergütungsstrukturen und limitierte Ressourcen gehemmt. Das Papier plädiert daher für eine schrittweise Einführung prähabilitativer Maßnahmen, orientiert an Risikogruppen und lokaler Versorgungsrealität. Es wird betont, dass Prähabilitation keine isolierte Maßnahme darstellt, sondern integrativ mit bestehenden Konzepten wie ERAS und Rehabilitation verknüpft werden sollte. Zudem adressiert das Papier die Notwendigkeit nationaler Vernetzung, gemeinsamer Standards, digitaler Lösungen sowie struktureller und politischer Unterstützung.

## Abstract (en)

Prehabilitation is gaining increasing importance as an integral component of modern, patient-centered perioperative care. The objective of this position paper is to outline the current national and international state of prehabilitation, to highlight existing barriers to its implementation and to provide practical recommendations for clinical application. Particularly in older, multimorbid and oncology patients, targeted preoperative interventions, such as exercise therapy, nutritional support, psychological counselling, and additional measures, can contribute to reducing postoperative complications, accelerating recovery and improving the quality of life. Despite the growing body of international evidence, implementation in Germany has so far been slow, often hindered by a lack of reimbursement structures and limited resources. This paper therefore advocates a stepwise introduction of prehabilitation measures, tailored to high-risk groups and aligned with local healthcare reality. It is emphasized that prehabilitation should not be regarded as an isolated intervention but as an integrative element within established concepts such as enhanced recovery after surgery (ERAS) and rehabilitation. Furthermore, the paper addresses the need for national networking, harmonized standards, digital solutions and structural as well as political support to ensure sustainable integration.

## Prähabilitation als Bestandteil moderner perioperativer Versorgung

Prähabilitation vor chirurgischen Eingriffen soll Patienten optimal vorbereiten, die physiologische Reaktion auf die Operation günstig beeinflussen und dadurch Genesung beschleunigen, Komplikationen verringern und Lebensqualität verbessern [1]. Da präoperative Defizite postoperativ schwer ausgleichbar sind, gewinnt das Konzept angesichts älterer, multimorbider Patienten zunehmend an Bedeutung. Ziel ist, beeinflussbare Risikofaktoren wie funktionelle Kapazität, Ernährungsstatus, Substanzmittelmissbrauch, psychische Belastung oder Anämie frühzeitig zu adressieren [2].

International hat die Prähabilitation in den letzten 10 Jahren an Relevanz gewonnen: PubMed listet fast 7000 Publikationen, darunter ca. 500 randomisiert kontrollierte Studien (RCTs) (Stand 06/2025). Positive Effekte werden trotz großer Heterogenität in den Metaanalysen bestätigt, v. a. für die Kombination von Ernährungs- und Bewegungstherapie [3, 4]. Nationale Leitlinien geben bislang nur zurückhaltende Empfehlungen, die aktuell in Arbeit befindlichen S3-Leitlinien werden neuere Daten stärker berücksichtigen [5]. Zudem wird betont, dass Prähabilitation nicht isoliert, sondern in Ergänzung zu ERAS und Rehabilitation zu sehen ist [5, 6].

In Deutschland ist die Umsetzung bisher träge, v. a. wegen fehlender Vergütungsstrukturen. Länder wie die Niederlande oder Spanien zeigen dagegen erfolgreiche Integration mit positiven klinischen und gesundheitsökonomischen Effekten [7].

Dieses Positionspapier soll einen aktuellen Überblick zur Prähabilitation, praxisnahe Empfehlungen formulieren und Barrieren sowie künftige Finanzierungs- und Kooperationsmodelle diskutieren:

1. Darlegen der Evidenz zu den Säulen der multimodalen Prähabilitation,
2. praxisnahe Empfehlungen zur Implementierung,
3. Diskussion künftiger Modelle zur Finanzierung und Zusammenarbeit.

## Säulen der Prähabilitation

### 1. Bewegungstherapie

Die Bewegungstherapie ist zusammen mit der Ernährungstherapie eine zentrale Säule der Prähabilitation. Ziel ist die Steigerung der funktionellen Kapazität vor Operationen, um Belastbarkeit, Muskelkraft und kardiovaskuläre Stabilität zu verbessern. So können physiologische Reserven genutzt und postoperative Komplikationen verringert werden [8]. Präoperative Defizite lassen sich postoperativ nur schwer ausgleichen, weshalb eine frühzeitige Intervention entscheidend ist.

Studien zeigen, dass Bewegungstherapie vor onkologischen Eingriffen den Funktionsverlust reduziert und kardiopulmonale Parameter verbessert und das Risiko für Komplikationen wie Pneumonien oder Thrombose senkt [9].

Netzwerkmetaanalysen bestätigen Vorteile von Ausdauertraining und inspiratorischem Muskeltraining (IMT) hinsichtlich Morbidität, Aufenthaltsdauer und Lebensqualität [10]. Eine narrative Übersicht kommt zu dem Schluss, dass Prähabilitation, kombiniert mit ERAS-Protokollen, die physiologische Reserve signifikant stärkt und postoperative Ergebnisse verbessert [11]. Besonders effektiv sind Programme mit Kombination aus Ausdauer‑, Kraft- und Atemtraining.

*Interventionsformen* (adaptiert an die allgemeinen Empfehlungen des American College of Sports Medicine) sind:

- *Krafttraining*: 2‑ bis 3‑mal/Woche, große Muskelgruppen, 60–80 % der Maximalkraft; Eigengewicht und Bänder als Ergänzung,
- *Ausdauertraining*: ≥ 3-mal/Woche für je 30 min in moderater Intensität (60–75 % der maximalen Herzfrequenz); Intervalltraining gilt als vielversprechend, aber noch nicht ausreichend evidenzbasiert [12],
- *Atemtherapie*: tägliche Übungen, besonders relevant bei COPD oder eingeschränkter Lungenfunktion [13].

Erforderlich ist eine enge Zusammenarbeit im therapeutischen und pflegerischen Team, insbesondere zwischen Physiotherapie, Sportwissenschaft und ärztlichem Team. Trainingspläne basieren auf initialer Diagnostik (z. B. 6‑Minuten-Gehtest, Sit-to-Stand, Spiroergometrie) und werden regelmäßig angepasst [11]. Digitale Lösungen und Telemedizin können Compliance und wohnortnahe Betreuung fördern.

Bewegung verstärkt die Wirkung anderer Interventionen: Ernährung verbessert Muskelstatus, psychologische Begleitung unterstützt Motivation. Patienten profitieren besonders von der Kombination [3].

### 2. Ernährungstherapie

Die medizinische Ernährungstherapie nimmt, wie auch die Bewegungstherapie, in der präoperativen Behandlung eine zentrale Position ein und ist wesentlicher Bestandteil der Prähabilitation [1].

#### Hintergrund zur Notwendigkeit einer strukturierten Erfassung des Ernährungsstatus.

Der Postaggressionsstoffwechsel (Stressstoffwechsel) nach großen chirurgischen Eingriffen zeichnet sich durch eine katabole Stoffwechselreaktion aus. Da der Körper in dieser Phase vorwiegend auf endogene Substrate zurückgreift, sind ausreichende Reserven entscheidend für die Fähigkeit zur Kompensation. Besonders die Proteinmangelernährung (krankheitsbedingter Gewichtsverlust, Muskelabbau, reduzierte Energie- und Proteinzufuhr) ist mit einem deutlich erhöhten postoperativen Mortalitätsrisiko vergesellschaftet.

Chronische Inflammationszustände, wie sie bei Tumorerkrankungen, chronisch entzündlicher Darmerkrankung (CED), Diabetes mellitus, Leberzirrhose oder Herzinsuffizienz auftreten, begünstigen die Entwicklung einer Mangelernährung. Betroffene benötigen daher eine individualisierte präoperative Ernährungstherapie, um Defizite gezielt auszugleichen.

**Abb. 1:**
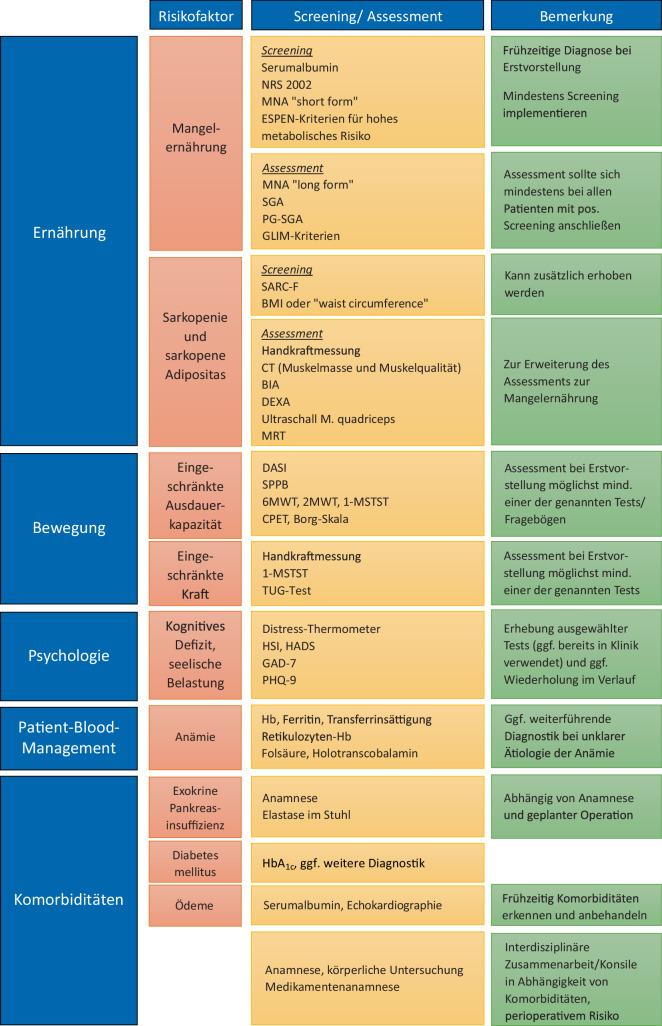
Übersicht über beispielhafte Maßnahmen zur Diagnostik von Risikofaktoren

Ein häufiges Missverständnis besteht darin, Mangelernährung ausschließlich mit Untergewicht oder einem niedrigen BMI gleichzusetzen. Auch Patienten mit normalem oder erhöhtem BMI können mangelernährt sein – insbesondere bei sarkopener Adipositas, die durch einen erhöhten Fett- und verminderten Muskelanteil gekennzeichnet ist. Diese Konstellation stellt einen eigenständigen Risikofaktor dar [14]. Daher sind ein differenziertes Screening und eine sorgfältige Beurteilung erforderlich, um gefährdete Patienten frühzeitig zu identifizieren (s. Abb. 1).

#### Durchführung einer medizinischen Ernährungstherapie im Rahmen der Prähabilitation.

Ziel der präoperativen Ernährungstherapie ist die bedarfsdeckende Versorgung mit Makro- und Mikronährstoffen. Bevorzugt erfolgt die Zufuhr über die orale Ernährung, ergänzt durch Supplemente, basierend auf den Empfehlungen der Deutschen Gesellschaft für Ernährung e. V. (DGE). Diese werden individuell angepasst, um metabolischen und krankheitsspezifischen Anforderungen gerecht zu werden.

Ein Schwerpunkt liegt auf der Deckung des erhöhten Proteinbedarfs, insbesondere bei chronischer Inflammation. Ziel ist es, den Verlust an Magermasse zu verhindern und einer Sarkopenie vorzubeugen. Der Proteinbedarf liegt bei 1,2–1,5 g/kg Körpergewicht/Tag, wobei individuelle Faktoren wie Nierenfunktion oder bestehende Sarkopenie berücksichtigt werden müssen [8, 15].

Der präoperative Einsatz immunmodulierender Ernährung (Omega-3-Fettsäuren, Arginin, Ribonukleotide) kann die Immunantwort positiv beeinflussen. Die aktuelle ESPEN-Leitlinie empfiehlt bei erhöhtem metabolischem Risiko eine Supplementation über 5 bis 7 Tage vor der Operation [15].

Mikronährstoffdefizite sollten bereits präoperativ überprüft werden. Bei unzureichender Versorgung über die Ernährung ist die Gabe eines Komplexpräparats (gemäß RDA) sinnvoll.

Bei manifester Mangelernährung ist ein strukturiertes ernährungstherapeutisches Assessment erforderlich. Dieses umfasst anthropometrische Messungen, Berechnung des individuellen Energie- und Proteinbedarfs sowie die Analyse der aktuellen Nahrungsaufnahme. Auf dieser Basis wird ein individueller Ernährungsplan erstellt [16].

Die Beratung sollte patientenorientiert erfolgen und soziale Aspekte – etwa die häusliche Versorgungssituation – einbeziehen. Konkrete Umsetzungshilfen und die Einbindung von Angehörigen fördern die Therapieadhärenz.

Reicht die normale Ernährung nicht aus, werden ergänzend orale Nahrungssupplemente (z. B. hochkalorische, proteinreiche Trinknahrung) eingesetzt. Bei unzureichender oraler Versorgung kann eine enterale oder parenterale Ernährung nach dem DGEM-Stufenschema erforderlich sein.

Die präoperative Ernährungstherapie erfolgt durch eine zertifizierte Ernährungsfachkraft [8, 16], die eng mit dem behandelnden ärztlichen Team zusammenarbeitet – insbesondere bei komplexer metabolischer Situation, Malassimilation oder Komorbiditäten. Ein Diabetes mellitus sollte präoperativ abgeklärt und ggf. eine diabetische Stoffwechsellage optimiert werden (Ziel HbA_1c_ < 8 %) [17].

Regelmäßige Verlaufskontrollen ermöglichen eine fortlaufende Reevaluation und Anpassung der Therapie [16]. Da entsprechende Strukturen noch nicht flächendeckend bestehen, können digitale Anwendungen oder regelmäßige telefonische Kontakte die persönliche Visite ergänzen.

### 3. Psychologische Intervention

Die Diagnose einer schweren oder lebensbedrohlichen Erkrankung stellt eine erhebliche psychische Belastung dar [18]. Das Ausmaß seelischer Reaktion hängt dabei von Resilienz, psychischer Komorbidität und sozialen Faktoren ab. Angst, depressive Symptome und geringe Selbstwirksamkeit beeinflussen das postoperative Outcome negativ [19].

In der onkologischen Therapie ist die Psychoonkologie seit Jahren integraler Bestandteil der multimodalen Behandlung. Sie unterstützt die Krankheitsverarbeitung, stabilisiert das psychische Befinden und verbessert die Lebensqualität [20]. Trotz digitaler Angebote bleibt die Implementierung psychologischer Interventionen in das präoperative Setting organisatorisch herausfordernd.

Studien zur Prähabilitation integrieren psychologische Maßnahmen regelmäßig in multimodale Programme. Eine randomisierte Studie bei Pankreaskarzinompatienten zeigte, dass bereits eine kurze präoperative psychologische Intervention signifikant zur Reduktion von Angstzuständen beitrug und die Selbstwirksamkeit der Teilnehmenden im Umgang mit Ängsten stärkte [21]. Eine Cochrane-Analyse bestätigt positive postoperative Effekte, bewertet die Evidenz wegen methodischer Heterogenität jedoch als gering [22].

Zur Erfassung psychischer Belastungen haben sich Screeningverfahren wie HADS, HSI oder das Distress-Thermometer bewährt [20]. Psychologische Interventionen – etwa Entspannungsverfahren, supportive Gespräche oder Coping-Strategien – fördern emotionale Stabilität und Krankheitsverarbeitung [18]. Ergänzend können digitale Gesundheitsanwendungen (DiGA) eingesetzt werden [20].

Ein enger interdisziplinärer Austausch zwischen Psychologie, ärztlichem Dienst, Ernährungs- und Physiotherapie erleichtert die Identifikation und Behandlung psychischer Belastungen. Niederschwellige Zugänge über andere Berufsgruppen fördern die Offenheit gegenüber psychotherapeutischen Angeboten.

### 4. Patient-Blood-Management

Patient-Blood-Management (PBM) steht für den verantwortungsvollen Umgang mit Blutprodukten. Obwohl Transfusionen lebensrettend sein können, sind sie mit erhöhter Morbidität, Infektionen und möglicherweise schlechterer Prognose bei Tumorerkrankungen assoziiert [23]. Bis zu 60 % der chirurgischen Patienten weisen präoperativ eine Anämie auf, meist aufgrund von Eisenmangel, der präoperativ effektiv durch intravenöse Eisengabe behandelt werden kann [24]. Das frühzeitige Erkennen und Behandeln einer Anämie ist zentral. Eine gezielte Diagnostik (Hb, Ferritin, Transferrinsättigung, Folsäure, B_12_) ermöglicht die spezifische Therapie. So lassen sich Transfusionsbedarfe reduzieren und das perioperative Risiko senken.

### 5. Nikotin- und Alkoholkarenz

Fortgesetzter Nikotin- oder Alkoholkonsum erhöht das Risiko postoperativer Komplikationen deutlich. Nikotin begünstigt pulmonale Komplikationen, stört die Mikrozirkulation und verzögert Wundheilung [25]. Übermäßiger Alkoholkonsum steigert das Risiko für Infektionen, Wundheilungsstörungen und postoperatives Delir, besonders bei älteren Patienten [26].

Die präoperative Beratung zur Abstinenz ist daher integraler Bestandteil der Prähabilitation und ist bereits in mehreren Studien zur multimodalen Prähabilitation untersucht. Eine kontinuierliche, engmaschige Begleitung ist wirksamer als kurze Einzelgespräche [27].

In der Praxis fällt die dauerhafte Umsetzung vielen Patienten schwer, insbesondere bei langjährigem Konsum. Aufklärung über die unmittelbaren Risiken kann jedoch zur kurzfristigen Abstinenz motivieren. Bei gelegentlichem Konsum reicht oft schon eine gezielte Information.

Da spezialisierte Programme oft fehlen, sind strukturierte Konzepte wie das 3‑wöchige BIÖG-Programm (Bundesinstitut für Öffentliche Gesundheit) zur Nikotinkarenz eine sinnvolle Ergänzung. Auch lokale Entwöhnungsangebote können genutzt werden.

## Wo fange ich mit der Prähabilitation an?

Die Vielzahl möglicher Interventionen kann auf den ersten Blick komplex wirken. Dennoch ist ein stufenweises Vorgehen sinnvoll und realistisch – auch bei begrenzten Ressourcen.

Zu Beginn sollte eine klar definierte Patientengruppe ausgewählt werden, bei der Prähabilitation eingeführt wird [28]. Parallel dazu sind ein multiprofessionelles Team (Chirurgie, Anästhesie, Physiotherapie, Ernährung, Psychoonkologie) sowie eine Projektleitung mit ausreichend zeitlichen Ressourcen erforderlich. Die Einbindung von Stakeholdern wie Pflege, Reha, niedergelassenen Partnern, Kostenträgern und Patientenvertretern ist entscheidend.

**Abb. 2:**
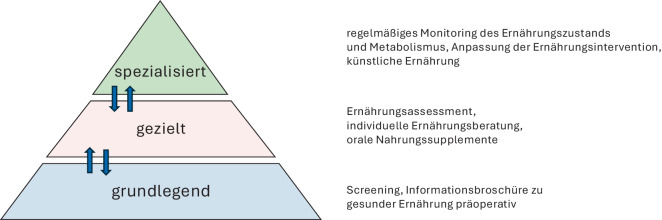
Aufbau Prähabilitation am Beispiel Ernährungstherapie. (Mod. nach Macmillan, the Royal College of Anaesthetists and the National Institute for Health Research Cancer and Nutrition Collaboration [29])

Bereits bestehende Programme und Konzepte, national wie international, können als Orientierung dienen. Entscheidend ist jedoch, dass deren Inhalte an die lokalen Gegebenheiten angepasst werden – mit Blick auf vorhandene Strukturen, Personalressourcen und Patientenpopulation. Zunächst genügt ein Basisprogramm mit den 3 Kernsäulen Bewegung, Ernährung und psychosoziale Unterstützung. Screening- und Assessmentinstrumente (Abb. 1) helfen bei der Auswahl geeigneter Maßnahmen. Im Verlauf können risikoadaptierte Module ergänzt werden. Kontinuierliche Evaluation und Qualitätssicherung sichern Wirksamkeit und Weiterentwicklung (s. Abb. 2 und 3).

**Abb. 3:**
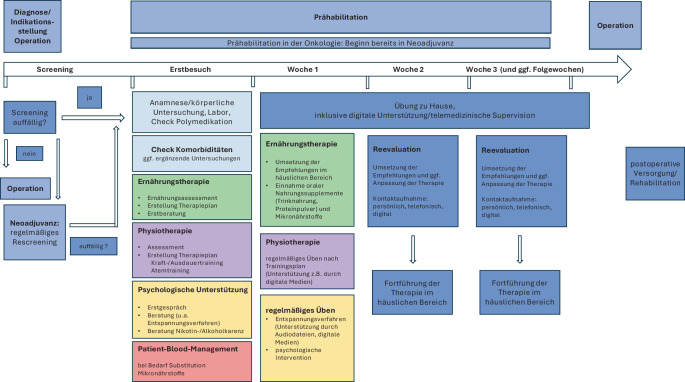
Übersicht und Beispiele zum Ablauf einer multimodalen Prähabilitation

## Indikationsspektrum und Fokussierung auf Hochrisikopatienten

Prähabilitation richtet sich an chirurgische Patienten, deren präoperative Optimierung das postoperative Ergebnis verbessern kann. Besonders relevant ist dies in der onkologischen Chirurgie, da viele Patienten bereits durch Erkrankung und multimodale Therapien belastet sind. Neben der Viszeral‑, Thorax-, gynäkologischen und urologischen Chirurgie profitieren auch Patienten in Herz‑, Gefäß- und komplexer Hernienchirurgie sowie der Endoprothetik [30, 31]. Jüngere Patienten mit chronisch entzündlichen Darmerkrankungen können ebenfalls durch Ernährung, psychologische Unterstützung und Kontrolle der Entzündungsaktivität profitieren [32].

Eine zentrale Querschnittsgruppe stellen Hochrisikopatienten dar. Einheitliche Kriterien fehlen, doch gelten Alter  > 65 Jahre, Mangelernährung, eingeschränkte funktionelle Kapazität, Gebrechlichkeit oder Komorbiditäten als wesentliche Risikomerkmale [8]. Die Etablierung klarer Definitionen ist ein wichtiges Entwicklungsfeld [8].

Empfohlen wird ein abgestuftes Vorgehen: Basismaßnahmen („universal prehabilitation“) für alle sowie intensivere Programme („targeted/specialised prehabilitation“) für Hochrisikogruppen. Individuell zugeschnittene Programme bieten hier das größte Potenzial zur Verbesserung des postoperativen Verlaufs.

## Wie viel Zeit braucht Prähabilitation – und wann ist sie möglich?

Ein entscheidender Aspekt ist der verfügbare Zeitraum vor der Operation. Für eine wirksame Prähabilitation gelten mindestens 3 Wochen als sinnvoll. Der tatsächliche Handlungsspielraum hängt jedoch stark von OP-Indikation und chirurgischem Kontext ab. Insbesondere bei onkologischen Patienten ist die Frage nach Wartezeiten komplex und entitätsspezifisch [33, 34]. In jedem Fall können die neoadjuvante Phase sowie die Pause zwischen Neoadjuvanz und Operation für prähabilitative Maßnahmen genutzt werden.

Grundsätzlich sollte Prähabilitation so früh wie möglich beginnen – idealerweise unmittelbar nach Feststellung der Behandlungsindikation [1]. Wartezeiten zwischen Diagnostik und Therapie können in der Regel genutzt werden, sofern keine dringliche Operation notwendig ist. In der Praxis stehen damit meist mindestens 2 bis 3 Wochen zur Verfügung.

## Beispiele und Implementierungsansätze in deutschen Kliniken

In Deutschland existieren bereits erste klinische Ansätze zur Implementierung von Prähabilitationsprogrammen. Diese zeigen erhebliche Heterogenität hinsichtlich ihrer inhaltlichen Struktur, organisatorischen Verankerung und verantwortlichen Profession. Die Spannbreite reicht von niederschwelligen edukativen Maßnahmen bis hin zu strukturierten, interdisziplinären, multimodalen Programmen.

In vielen Kliniken beginnt Prähabilitation mit einer standardisierten Patientenedukation im Rahmen der präoperativen Vorbereitung. Diese umfasst häufig

- systematische Ernährungsempfehlungen, insbesondere zur Einnahme protein- und energieangereicherter Trinknahrungen,
- die Ausgabe einfacher Hilfsmittel wie Atemtrainer,
- die Anleitung zu einer aktiven Lebensweise mit Bewegung und Atemübungen.

Zur Unterstützung kommen dabei häufig schriftliche oder visuelle Materialien (z. B. Flyer oder standardisierte Informationsbögen) zum Einsatz. Diese Form der Prähabilitation ist ressourcenschonend, leicht implementierbar und kann unabhängig von spezialisierten Strukturen erfolgen.

Darüber hinaus haben einige Zentren spezialisierte prähabilitative Sprechstunden und Programme etabliert, die eine systematische Erfassung von Risikofaktoren und eine interprofessionelle Betreuung erlauben. Auf dieser Basis erfolgt eine gezielte Beratung durch qualifizierte Ernährungsfachkräfte sowie eine Anleitung oder Initialisierung eines körperlichen Trainingsprogramms durch Physiotherapeuten. Solche Sprechstunden ermöglichen eine individualisierte und evidenzbasierte Planung präoperativer Interventionen, erfordern jedoch eine klare organisatorische Verankerung und interprofessionelle Zusammenarbeit.

In der München Klinik Neuperlach koordiniert beispielsweise die Stabsstelle Ernährungsmedizin den Ablauf der Prähabilitation. Im interdisziplinären Ablauf der Abteilungen Gastroenterologie, Onkologie und Viszeralchirurgie wurden relevante Screening-Tools systematisiert, welche eine frühzeitige Identifizierung von Patienten erlauben, die einer Prähabilitation bedürfen: (1) Screening auf Mangelernährung bei Erstaufnahme, (2) Bestimmung von Serumalbumin und Eisenstatus bei allen viszeralonkologischen Patienten, (3) routinemäßige Mitbestimmung des Sarkopenie-Index im Rahmen des Stagings CT-Abdomen, (4) ernährungsmedizinisches Konsil zur Einschätzung des Prähabilitationsbedarfs, (5) Vorstellung in Tumorboard mit Empfehlung.

Für eine nachhaltige Implementierung der Prähabilitation ist es entscheidend, frühzeitig klare Verantwortlichkeiten zu definieren und ein interdisziplinäres Kernteam zu etablieren. Es empfehlen sich eine regelmäßige Reevaluation der hausinternen Abläufe sowie eine kontinuierliche Schulung von ärztlichem Dienst und Pflege, um die Compliance zur Durchführung der Maßnahmen hoch zu halten. In Zukunft werden digitale Anwendungen auch hier eine größere Rolle spielen und sind in Deutschland zum Teil schon auf dem Markt bzw. in der Entwicklung oder Evaluierung.

Aktuelle deutsche Forschungsprojekte beleuchten weitere Implementierungsaspekte:

- Multizentrische Studien: Das PRAEP-GO-Trial untersucht prähabilitative Programme bei älteren (prä)frailen Patienten vor orthopädischen Eingriffen, wobei qualitative Daten zu Barrieren und förderlichen Faktoren der Teilnahme erhoben werden [35].
- Studienprotokolle:
  - „PRECOVERY“: Diese groß angelegte, randomisierte Studie adressiert prähabilitative Interventionen bei älteren Patienten vor elektiven kardiologischen Eingriffen und integriert multimodale Module (körperliches Training, psychosoziale Komponenten, Ernährung etc.) [36].
  - Einen innovativen Ansatz verfolgt die LUMOS-Studie, eine im Deutschen Register Klinischer Studien (DRKS00033612) registrierte, multizentrisch randomisierte kontrollierte Studie. Sie untersucht die Effektivität eines strukturierten, App-basierten Prähabilitationsprogramms im häuslichen Umfeld im Vergleich zu einer rein edukativen digitalen Standardversorgung. Die LUMOS-Studie ist insbesondere aus implementierungswissenschaftlicher Perspektive relevant, da sie digitale Technologien gezielt nutzt, um strukturelle Hürden der Prähabilitation – etwa begrenzte personelle Ressourcen, fehlende wohnortnahe Angebote oder eingeschränkte Mobilität der Patienten – zu adressieren.

Insgesamt verdeutlichen diese Forschungsansätze, dass sich die Prähabilitation in Deutschland zunehmend von einzelnen, lokal initiierten Projekten hin zu systematisch evaluierten, wissenschaftlich fundierten Versorgungskonzepten entwickelt. Die Ergebnisse laufender Studien werden entscheidend dafür sein, ob und in welcher Form Prähabilitation künftig als fester Bestandteil präoperativer Behandlungspfade etabliert werden kann.

## Zukünftige Modelle und Kooperationsmöglichkeiten

Trotz positiver Evidenz bleibt die Umsetzung von Prähabilitation in Deutschland begrenzt. Hauptbarrieren sind fehlende Strukturen, mangelnde Abrechnungsmöglichkeiten und die unzureichende Integration in standardisierte Versorgungspfade. Modellprojekte zur Prähabilitation berücksichtigen diese Herausforderungen wie das PRECOVERY-Projekt der Universität Göttingen [31].

Digitale Anwendungen innerhalb der Prähabilitation gewinnen zunehmend an Bedeutung sowohl in Forschung als auch in der klinischen Praxis. Sie ermöglichen eine ressourcenschonende Umsetzung, stärken das Patient-Empowerment und reduzieren den Bedarf an Klinikbesuchen – ein Vorteil insbesondere für ältere oder mobilitätseingeschränkte Patienten. Studien belegen die Machbarkeit, Sicherheit und Nichtunterlegenheit digitaler Konzepte [37].

Ein Erfolg versprechender Ansatz ist der Aufbau regionaler Netzwerke, in denen Kliniken Ressourcen bündeln und Prähabilitation zentral anbieten. Auch die Einbindung in bestehende Strukturen wie Tageskliniken, onkologische Zentren oder die ambulante spezialfachärztliche Versorgung (ASV) ist möglich und wird teils bereits umgesetzt.

Langfristig sollten Daten zur Prähabilitation systematisch in Qualitätssicherungsregister einfließen. Auch die Nutzung digitaler Routinedaten aus elektronischen Patientenakten kann helfen, Wirksamkeit und Effizienz kontinuierlich zu bewerten.

## Fazit

Prähabilitation ist ein naheliegendes und zunehmend diskutiertes Konzept, dessen Umsetzung jedoch durch heterogene Evidenz und fehlende Finanzierungsstrukturen gebremst wird.

Zentrale Erfolgsfaktoren sind interdisziplinäre und multiprofessionelle Zusammenarbeit sowie die Definition standardisierter Core-Outcomes, die Studien vergleichbar machen. Auch die koordinierte Entwicklung digitaler Unterstützungsangebote ist notwendig.

Für eine nachhaltige Implementierung braucht es enge Vernetzung: national zur Organisation der Versorgung, international für die Weiterentwicklung der Evidenz, Fachgesellschaften wie die DGAV, indem sie Expertise bündeln, Projekte fördern und konsensbasierte Empfehlungen entwickeln. Gemeinsam mit Verbänden, Kostenträgern und Politik sollten sie die langfristige Verankerung prähabilitativer Leistungen in der Regelversorgung und deren Abbildung in Abrechnungsmodellen vorantreiben.
